# Space as a mental toolbox in the representation of meaning

**DOI:** 10.1098/rsos.240985

**Published:** 2024-11-06

**Authors:** Natalia Zarzeczna, Tisa Bertlich, Bastiaan T. Rutjens, Ida Gerstner, Ulrich von Hecker

**Affiliations:** ^1^ University of Essex, Colchester, UK; ^2^ Johannes Gutenberg-Universität Mainz, Mainz, Germany; ^3^ University of Amsterdam, Amsterdam, The Netherlands; ^4^ Universität Mannheim, Mannheim, Germany; ^5^ Cardiff University, Cardiff, UK

**Keywords:** meaning, coherence, logical reasoning, spatial representation, abstract concepts

## Abstract

The experience of meaning has been found to be mapped onto spatial proximity whereby coherent—in contrast to incoherent—elements in a set are mentally represented as closer together in physical space. In a series of four experiments, we show that spatial representation of coherence is malleable and can employ other meaningful concrete dimensions of space that are made salient. When given task instructions cueing verticality, participants represented coherence in the upper vertical location when making judgements about the logical validity of realistic (Experiments 1 and 4) and unrealistic syllogistic scenarios (Experiment 3). When the task instruction made the spatial proximity between the stimuli materials and the participant salient (subjective proximity), participants represented coherence as spatially close to themselves (Experiment 2). We also found that being accurate in judging the validity of syllogisms was associated with representing coherence in the upper visual field or close to oneself. Overall, our findings show that identifying semantic links between an abstract concept and a given spatial dimension involves using that dimension to create spatial metaphoric mappings of the concept.

## 1. Introduction

Much of human thought concerns understanding ideas that cannot be directly experienced. Indeed, one of the basic evaluations that people make is deriving meaning from an integrated set of abstract ideas, or in other words, making judgements about their coherence. Abstract ideas are inherently intangible, detached from perception, and do not have a clear referent in the physical world. Unlike concrete concepts, abstract ones are more challenging to understand, and people tend to disagree more about their definitions (see [1], for a review). Many theoretical perspectives have been proposed to illustrate the mental representation of abstract concepts. Embodied views suggest that abstract concepts are grounded in sensorimotor experiences [2,3], while other theories propose amodal linguistic representations combined with embodiment [4], or multiple representations involving modal, linguistic, cultural and social influences [1,5,6]. Further, construal level theory illustrates the fact that people have the ability to think about abstract objects that cannot be directly experienced in the here and now by using psychological distance dimensions [7]. Regardless of whether abstract concepts are always grounded in sensorimotor experiences, amodal linguistic symbols, a combination of all these processes or the use of psychological distance, there is robust evidence suggesting that the mental representation of abstract concepts links with physical space, as consistent with the assumptions made by conceptual metaphor theory [8,9].

### 1.1. Space as a mental toolbox

Within embodiment accounts, such as conceptual metaphor theory, it has been suggested that abstract concepts are metaphorically tied, in long-term memory connections, to specific spatial dimensions through early sensorimotor experiences with physical space. These experiences are later transformed into perceptual symbols to be used in mental representations. For example, Schubert [10] argued that power is conflated with verticality through early experiences, whereby children learn that their parents or older siblings, who are taller than them, are also more powerful. This acquired association between power and verticality is later transmitted and reinforced through language and cultural artefacts. Further, von Hecker and Hahn [11] postulated that coherence is grounded in a linguistic metaphor, whereby elements that make sense are perceived as close together. In the current research, we challenge such metaphoric grounding accounts by proposing that spatial mapping of abstract concepts can occur in an *episodic* manner as long as people’s attention is guided towards a particular spatial dimension and that spatial dimension can be meaningfully utilized to reason about abstract concepts. Specifically, we argue that spatial dimensions are a flexible mental ‘toolbox’ in reasoning that can be used in adaptive ways that are appropriate for a given task. In fact, although it has been suggested that linking power with vertical space is an innate proclivity [12], such embodiment of power is then subjected to individual learning. Consistent with this, recent literature suggests multidimensionality in the representation of all concepts, with certain kinds of abstract concepts being tied to sensorimotor experiences, but also grounded in sociality, language and individual differences [6,13,14]. Similarly, in our view, it is unlikely that spatial dimensions as such have *a priori*, long-term memory metaphoric links with specific abstract concepts. Rather, these metaphoric mappings are created in an episodic manner under appropriate environmental conditions.

Previous research indirectly aligns with these theoretical points: While many abstract concepts might be linked metaphorically with physical space (e.g. power/valence/auditory pitch/numbers to verticality [10,15–18]), other research found that magnitude dimensions (dominance, age, wealth), which are also spatially mapped (onto the horizontal dimension), do not necessarily have explicit metaphoric links with space (see [16] for a similar argument when it comes to the representation of auditory speech being linked to space, mass or visual lightness). Rather, such magnitude dimensions are more likely grounded in reading/writing direction and to be represented to the left in cultures with left-to-right writing habits [19]. Similarly, the cross-talk between action and abstract language differs depending on culture, whereby only in an Italian but not a Farsi sample of participants, language and action (specifically gestures) seem integrated, with gesturing facilitating processing of concrete sentences. This is possibly due to a tighter association between gesture and language among Italians than Farsi individuals [20].

In light of this evidence, we suggest that spatial mapping involves an interaction between the cognitive spatial toolbox and a particular task setting. For this reason, we investigate spatial correlates of coherence on the vertical dimension and as spatial proximity.^1^ If the experimental task affords verticality, then coherence would be mapped onto a particular vertical spatial cue. When the task affords spatial proximity, then coherence would be mapped onto a particular proximity cue. The choice of an appropriate spatial cue within the available spatial dimension would then be guided by episodically created semantic links relevant to the mapped abstract concept, likely based on learning history.

### 1.2. Mental representation of coherence

Coherence, as a general concept in logic and philosophy, describes the impression that a number of propositions overlap, are relevant to each other, or mutually support and corroborate each other. The term does not refer to the individual propositions themselves, but to the degree to which the propositions ‘make sense’ as an entire set in logical ways [21–24]. Coherence has also been conceptualized as a fundamental human need and a key facet of meaning in life [25,26] (Martens & Rutjens 2023, unpublished data). Specifically, perceptions of expected consistency (based on learning history) between elements in the world contribute to increased meaning in life. Preserving meaning is so important that people strive to compensate for threats to meaning that has large implications for physiological reactions [27,28], intergroup processes [29,30] or religious beliefs [31,32].

Further, examining coherence provides an opportunity to study high level of abstractness. Previous literature focused on individual abstract concepts and their mental representations (e.g. power, morality, valence; see below for an overview of this literature). Unlike individual abstract concepts, coherence refers to the ensemble of all premises simultaneously concerned. In this sense, coherence is a process of abstraction that represents a higher abstractness level compared to its premises ([33]; see also e.g. [34]).

Consistent with semantic network theories, suggesting that semantically related concepts are spatially organized in close proximity in memory (e.g. [35]), previous research found that the extent to which concepts ‘fit’ together are also spatially represented together: specifically, it has been demonstrated that coherence can be mapped onto proximity in mental space [11]. In this research, a diversity of stimulus materials (e.g. balanced versus unbalanced Heiderian triads of social relations among three persons,^2^ or categorical syllogisms) were used to operationalize coherence. When participants were presented with coherent versus incoherent concepts, they tended to see a gap presented between the concepts as narrower.

However, there are also reasons to believe that coherence (versus the lack thereof) might be linked to perceptions of high (versus low) verticality. First, one tenet of construal level theory [7] is that high-level construals involve abstract processing, such as coherent representations based on relations between more fundamental concepts (e.g. the experience of a ‘fit’ between the two premises and the conclusion of a syllogism). In this view, low-level construals are associated with concrete processing that concerns basic, specific and detailed features of concepts. This also includes a situation where these basic pieces of information (propositions) cannot be ‘fitted’ into a coherent overall representation.

Second, and starting from the above assumptions, Slepian *et al*. [36] ventured a semantic interpretation of the metaphorical mapping between coherence and verticality. High verticality might be associated with high perceptual scope; that is a spatial location from where it is easier to (mentally) see the overall meaning of a number of ideas and how these ideas can fit together. When participants experienced high versus low verticality, they were more likely to see high concept inclusivity (e.g. the word camel belonging to the category of vehicle). Consequently, if abstract processing styles are associated with high simulated space and increased coherence [7,37], then it is likely that the perception of coherence might also involve a placement on the vertical dimension.

A third reason why coherence might be mapped onto verticality comes from research on metaphoric mappings with related semantics. With regards to logical reasoning tasks like syllogisms, in common language, the term ‘valid’ (in contrast to ‘invalid’) implies aspects of power (powerful–powerless) and quality (good–bad) that involve semantically involved dimensions, as such people show associations between powerful individuals and upper vertical positions [10]. Further, male but not female targets who are seen as powerful are simulated at high vertical positions, suggesting that relevant social knowledge (i.e. gender stereotypes) can contribute to such representations [38]. Likewise, the vertical dimension can be used to represent valence (good/up versus bad/down) [17,39–41]. Overall, in the present context, we argue that semantic connotations between coherence (as valid, good, stable, robust, etc.) and other abstract concepts with known potential for vertical representations in mental space may facilitate a corresponding vertical representation for the coherence concept itself.

### 1.3. Flexibility of metaphoric mappings in individual abstract concepts

The above evidence suggests that metaphoric mappings are flexibly guided by social knowledge. In a related vein, culture has also been shown to be important in determining spatial representations [19,42,43], while recent work demonstrates that concepts can be mapped onto multiple spatial dimensions, e.g. at around 5 years of age, children begin to show a preference for representing temporal events from left-to-right, but also top-to-bottom ([44,45]; also see [46,47]). We extend this literature by focusing on coherence because this experience is distinct from other previously studied magnitude representations (power, dominance). In such representations, a position on the horizontal dimension symbolizes a certain degree of magnitude (e.g. the most dominant element is represented to the left in cultures with left-to-right reading/writing habits; [19). As coherence representations do not address the magnitude of a concept but the relatedness between concepts, the representation of coherence cannot be directional as a mapping of quantity.

We suggest that coherence, similar to magnitude dimensions, does not employ a long-term memory and metaphoric one-to-one spatial mapping (e.g. coherent elements are grounded in spatial proximity, [11]) under all circumstances (see also [48]). Instead, the representation of coherence can flexibly switch in an episodic manner to alternative mappings as long as these mappings are meaningfully linked to the content [49,50]. In this sense, one may view metaphoric mappings as an episodic result of processes in working memory, whereby mental models about the abstract concept dimension are related to a target physical dimension, according to which particular physical dimension is made salient, or afforded, by the experimental constraints and its instructions [51,52].

As such, we postulate that the cognitive system can utilize *a dimension* of space for mappings of abstract concepts depending on which spatial cues are available in the environment. Subsequently, we suggest that particular, semantically relevant, spatial cues (upper vertical position/subjective proximity from the self in the case of coherence) are then consistently allocated to represent a particular abstract concept, creating episodic metaphoric links with that concept. That is, we suggest flexibility in spatial dimensions, but the polarity (upper vertical location/spatial closeness) would be consistently chosen based on intuitive mapping likely shaped by learning history.

We predicted that, provided suitable experimental conditions, coherence will be represented on the vertical dimension, with the experience of coherence involving upper vertical positions and experience of lack of coherence involving lower vertical positions. However, if experimental conditions afford a proximity mapping, then coherence would be mapped onto proximity in mental space [11]. This means to also assume that abstract reasoning is not grounded in a previously acquired and symbolically linked physical spatial dimension used for mapping a particular concept, but rather reasoning may use any concrete dimension that is best available in the given context to meaningfully facilitate processing [1,52,53].

### 1.4. Present research

In the current work, we operationalize making sense as the experience of coherence. To broadly assess coherence, we use two types of syllogistic tasks. The first type focuses on reasoning about content and expected relations between the premises ([25]; see table 3 for examples). The second type focuses on the logical form of the premises beyond content ([11]; see table 5). Altogether our materials assess coherence as reasoning about expected associations as well as strict logical form of arguments. We use syllogistic materials, whereby the two premises of a syllogism represent two propositions containing basic information, whereas the conclusion represents a third proposition, expressing either a consequence that either makes sense or does not make sense based on the basic information, thereby incorporating either coherence or lack of coherence, respectively, with regards to the entire set of propositions. We tested these ideas across Experiments 1–4 by presenting participants with syllogisms that either made sense (coherent) or did not make sense (incoherent; see table 1 for details). In all experiments, participants were asked to think about whether each syllogism made sense or not and then complete a spatial task, whereby they indicated the vertical position or spatial proximity (in relation to the self, i.e. subjective proximity) of target words, which represented key concepts from the syllogism. Specifically, as the key concept or expression covers the conclusion of the syllogism, its meaning is most sensitive to whether the syllogism is experienced as coherent or not. Therefore, any mapping of the meaning of the conclusion, onto space, is indicative of mapping of coherence onto space. We measured the proportions of times participants selected each vertical and proximity position (upper/lower and far away/close) for coherent and incoherent target words and how accurate participants were in judging whether the syllogisms made sense or not.

**Table 1.** Design across experiments.

	experiment 1 (laboratory)	experiment 2 (online)	experiment 3 (laboratory)	experiment 4 (online)
coherence manipulation	syllogisms about gene editing framed in psychologically close or distant ways	syllogisms about gene editing	abstract syllogisms with unbelievable content	syllogisms about gene editing
spatial task	pathway drawing	pathway drawing	no drawing	no drawing
spatial task instructions	detect whether the target was presented ‘above’ or ‘below’ the midpoint of the previously presented circle.	detect whether the target was presented ‘close’ to or ‘far’ away from you.	detect whether the target was presented ‘above’ or ‘below’ the midpoint of the previously presented circle.	detect whether the target was presented ‘above’ or ‘below’ the midpoint of the previously presented circle.
response mode	keyboard arrows ‘up’ and ‘down’ (vertical mode)	keyboard keys ‘d’ and ‘j’ counterbalanced (horizontal mode)	keyboard arrows ‘up’ and ‘down’ (vertical mode)	keyboard arrows ‘up’ and ‘down’ (vertical mode)
dependent variable	validity accuracy; the proportion of ‘above’ and ‘below’ responses	validity accuracy; the proportion of ‘close’ and ‘far’ responses	validity accuracy; the proportion of ‘above’ and ‘below’ responses	validity accuracy; the proportion of ‘above’ and ‘below’ responses

As an additional aim, we explored whether spatial representation of coherence involved improved performance in decision-making about the coherence of syllogisms. Specifically, we investigated whether participants’ performance in judging the validity of syllogisms across all experiments was increased if participants selected the spatial location semantically relevant to coherence, that is the upper vertical and the close spatial location for coherent, and the lower vertical and the far spatial location for incoherent syllogisms.

## 2. Experiment 1: coherence as verticality and psychological distance

In Experiment 1, we examined whether coherence would be mapped onto the vertical dimension. As an additional aim, we tested whether psychological distance [7] would interact with a potential vertical mapping of coherence. In their design, Bar-Anan *et al*. [54] had combined the template pathway drawing with a proximity-salient instruction, as participants had to judge the spatial proximity of words (in terms of the drawing: close or far) that were presented at either high or low locations within the drawing. Participants were faster to correctly indicate spatial proximity (*sensu* drawing) of a word when its psychological distance in terms of its meaning (e.g. friend/close or enemy/far) matched the spatial proximity. To this end, the stimulus sentences we used in Experiment 1 indicated psychological closeness or high distance. If there was an effect of psychological distance (and coherence represented vertically), this would mean either a main effect of psychological distance, or an interaction of psychological distance with the coherence factor. Specifically, such interaction should take the shape of participants exhibiting a stronger response bias to say ‘above’ (as indicating the target position relative to the drawing’s midpoint) for coherent trials if they contained a ‘distant’ sentence as compared to coherent trials containing a ‘close’ sentence. Likewise, one would expect a stronger tendency to say ‘below’ for incoherent trials if they contained a ‘close’ sentence, as compared with incoherent trials containing a ‘distant’ sentence. Such findings would suggest that mental representations can use diverse spatial cues (proximity and verticality) simultaneously insofar as the cues are meaningful in respect to the projected concepts and task instructions [47].

Based on the proportion of participants’ choices of ‘above/below’ responses and the objective positions of the words, signal detection parameters *d*′ and *c* [55] could be computed. We hypothesized that if participants represented coherence at the top, they would have a general tendency (as captured by parameter *c*) to respond ‘above’ more to the targets that came from coherent versus incoherent syllogisms. Finally, we predicted an interaction between coherence and psychological distance to manifest in a more pronounced tendency to respond ‘below’ to targets from incoherent syllogisms combined with close framing, as compared with targets from incoherent syllogisms combined with distant framing. We also predicted a more pronounced tendency to respond ‘above’ to targets from coherent syllogisms combined with distant framing, as compared with targets from coherent syllogisms combined with close framing. If these predictions were borne out, this would then support the idea that both coherence and psychological distance might jointly contribute their respective metaphoric mappings (one onto proximity, one onto verticality) to the generation of their responses.

### 2.1. Method

#### 2.1.1. Transparency and openness

The data collection took place between 2021 and 2023. Raw data, analysis code and presentation software materials are available on OSF. Where applicable, we provide pre-registration links. The findings are confined to the context of Western Europe.

#### 2.1.2. Participants

We recruited 61 participants from a population of university students in the UK (see table 2 for details). We estimated the sample size based on a power analysis and using a range of effect sizes for a paired samples *t*‐test: Cohen’s *dz =* 0.35–0.40, α = 0.05, 80% power, using the G*Power software. We selected these effect sizes based on previous research on spatial representation of stereotypic information ([38]; *dz =* 0.35; *n* = 60–78; 6 experiments) and on spatial representation of coherent information ([11]; *dz =* 0.40; *n* = 24–60; 4 experiments). The estimated required sample size was between 52 and 67 participants. All studies reported in this manuscript received university ethics committee approvals. All materials, data and analysis codes for all experiments are available on OSF: https://osf.io/8uxym/.

**Table 2.** Participants’ demographics.

	experiment 1 (laboratory)	experiment 2 (online)	experiment 3 (laboratory)	experiment 4 (online)
*N*	61	113	70	118
age (years)	*M =* 20.03, s.d. *=* 3.68, range: 18–43	*M =* 20.01, s.d. *=* 2.93, range: 18–35	*M =* 19.54, s.d. *=* 0.94, range: 18–22	*M =* 20.72, s.d. *=* 3.44, range: 18–47
gender	women: 54, men: 5, non-binary: 1, prefer not to say: 1	women: 93, men: 15, non-binary: 4, missing: 1	women: 61, men: 7, non-binary: 2	women: 80, men: 34; non-binary: 3, missing: 1
English fluency (1—not fluent at all, 7—very fluent)	*M =* 6.56, s.d. *=* 0.91	*M =* 6.46, s.d. *=* 0.73	*M =* 6.66, s.d. *=* 0.95	*M =* 6.36, s.d. *=* 0.85

#### 2.1.3. Design and materials

#### Coherence and psychological distance syllogisms

We used a mixed design in which coherence was manipulated within-participants and psychological distance was manipulated between-participants. We operationalized coherence by presenting participants with syllogisms that either made sense (i.e. coherent) or did not make sense (i.e. incoherent). We created the materials ourselves. Each syllogism discussed a gene editing technique used in humans (e.g. Talen, CRISPR/Cas9, see table 3). In each syllogism, the first two sentences were the premises (e.g. ‘I might need cell-based therapies at some point in my life. Zinc-finger nucleases are used to develop genetic cell-based therapies’), while the last one was the conclusion (e.g. ‘I may benefit from Zinc-finger nucleases-based therapies’). A syllogism where the conclusion followed directly from the premises was classified as coherent, while a syllogism where the conclusion did not follow from the premises was classified as incoherent. To manipulate whether the syllogisms made sense or not, we modified the second premise in each syllogism, such that it was either relevant (in coherent syllogisms) or irrelevant (in incoherent syllogisms) to the first premise and the conclusion. Each participant saw five coherent and five incoherent syllogisms in each block of trials.

**Table 3.** Materials based on content in Experiment 1.

coherent-close [distant]	incoherent-close [distant]	target word
I might need cancer treatment at some point in my life.	I might need cancer treatment at some point in my life.	CRISPR/Cas9
CRISPR/Cas9 helps to develop new, effective cancer treatments.	CRISPR/Cas9 helps to develop new, effective ways to treat muscular dystrophy.	
**CRISPR/Cas9-based treatments will become available in local UK [USA] hospitals within the next 2 [10] years**.	**CRISPR/Cas9 based treatments will become available in local UK [USA] hospitals within the next 2 [10] years**.	
CRISPR/Cas9 may help me.	CRISPR/Cas9 may help me.	
At some point, I might benefit from medical research, but the government has only a limited amount of money to spend.	At some point, I might benefit from medical research, but the government has only a limited amount of money to spend.	CRISPR/Cas9
CRISPR/Cas9 is a cheap and effective method used in medical research.	CRISPR/Cas9 is a cheap and effective method used in research on food shortage problems.	
**CRISPR/Cas9 will be used in research centres all over the UK [USA] to improve medical research within the next 2 [10] years**.	**CRISPR/Cas9 will be used in research centres all over the UK [USA] to improve medical research within the next 2 [10] years**.	
CRISPR/Cas9 may be beneficial to me.	CRISPR/Cas9 may be beneficial to me.	
I might need cell-based therapies at some point in my life.	I might need cell-based therapies at some point in my life.	Zinc-finger nucleases
Zinc-finger nucleases are used to develop genetic cell-based therapies.	Zinc-finger nucleases are used to develop animal disease models.	
**Zinc-finger nucleases-based therapies will become available in local UK [USA] hospitals within the next 2 [10] years**.	**Zinc-finger nucleases-based therapies will become available in local UK [USA] hospitals within the next 2 [10] years**.	
I may benefit from Zinc-finger nucleases-based therapies.	I may benefit from Zinc-finger nucleases-based therapies.	
I might need newly developed drug therapies.	I might need newly developed drug therapies.	Zinc-finger nucleases
Zinc-finger nucleases help to test the safety of new genetic drug therapies.	Zinc-finger nucleases help to test efficiency of genetic engineering in mice.	
**Zinc-finger nucleases-based drug therapies will become available in local UK [USA] hospitals within the next 2 [10] years**.	**Zinc-finger nucleases-based drug therapies will become available in local UK [USA] hospitals within the next 2 [10] years**.	
Zinc-finger nucleases ensure the safety of my drug therapies.	Zinc-finger nucleases ensure the safety of my drug therapies.	
I might develop a genetic disease.	I might develop a genetic disease.	Talen
Talen can be utilized to correct the genetic errors that underlie genetic diseases.	Talen can be utilized to develop tools for the production of biofuels.	
**Talen will be used in research centres all over the UK [USA] to understand various diseases within the next 2 [10] years**.	**Talen will be used in research centres all over the UK [USA] to understand various diseases within the next 2 [10] years**.	
I may benefit from Talen use.	I may benefit from Talen use.	

To manipulate psychological distance, we added one sentence to each syllogism just before the conclusion. The sentence referred to whether the discussed gene editing technique was available in the UK (the psychologically close condition) or in the USA (psychologically distant condition; see table 3 for all syllogisms).

#### Spatial task

**Figure 1.**
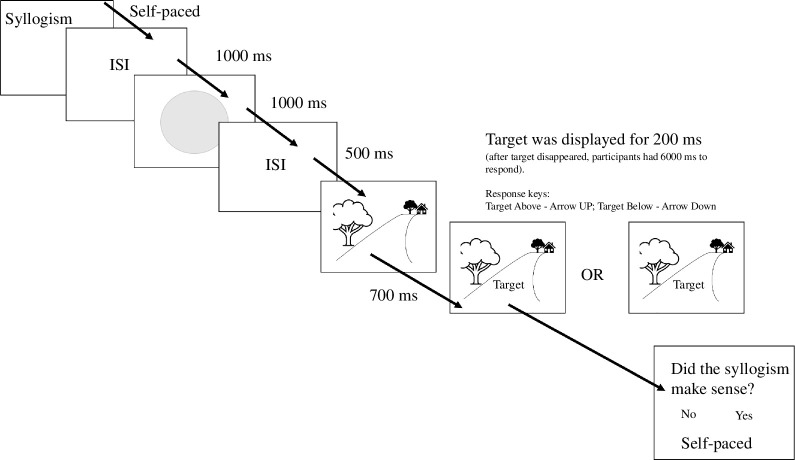
Trial events and timing (min) in Experiment 1. The target word presented in each trial was displayed either slightly above or below the midpoint and was superimposed onto the pathway. When the target appeared on the screen, participants indicated whether they thought the target was above or below the midpoint, with 6000 ms time limit. Upon completion of the spatial task, participants indicated whether the syllogism made sense or not (with unlimited time to respond).

After reading a single syllogism in each trial, participants completed the spatial task. In this task, we first presented participants with a grey circle in the middle of the screen followed by a picture representing a pathway. The picture was based on Bar-Anan *et al*.’s (2007) design (see figure 1). We used it to simulate perception of depth, such that participants would perceive spatial closeness in the lower half, or spatial distance in the upper half of the drawing. Then, we presented a target word superimposed onto the drawn pathway. Participants’ task was to quickly and accurately judge whether the target word was presented ‘above’ or ‘below’ the midpoint of the grey circle presented earlier by pressing the arrow up for ‘above’ and down for ‘below’ on the computer keyboard. At the end of the trial, participants indicated whether the syllogism made sense or not (see figure 1 for details). They pressed the arrow pointing left if they thought the syllogism did not make sense or the arrow pointing right if the syllogism made sense. The presentation order of the syllogisms was randomized within each block. Across all experiments, the events in each trial were explicitly explained to participants with written instructions. We informed participants that first they would read a syllogism and judge whether it made sense or not without immediately providing a response. Then, they would complete a spatial task, whereby they would see a target word/expression taken from the syllogism they had read before, and they would need to judge its spatial position. We also informed participants that the task would be difficult as the spatial position would vary only slightly, and they should make their best guess if they could not clearly see the position.

The spatial location of stimuli and their size on the screen were specified in height units, whereby the middle of the screen was defined as: *x*-axis: 0, *y*-axis: 0, top-right of the screen is equal to: 0.8, 0.5, while bottom-left: −0.8, −0.5. The height units are relative to the height of the computer window (not the screen height), such that the stimuli position and size were scaled to the window size and hence would remain proportionate across different window sizes. The circle size was 0.5. The target word size was 0.03 and its horizontal position was always set to 0, while the position above or below the midpoint was randomly assigned in each trial to be either −0.002 (below the midpoint) or 0.002 (above the midpoint). We varied the position of target words only slightly to make it difficult for participants to judge the objective spatial position (all experimental software files are available on OSF).

#### Procedure

Participants completed the experiment in the laboratory. They were sat around 60 cm away from the computer screen. All stimuli were presented using the open-source software PsychoPy v. 2023.2.3 [56]. Before starting the experiment, we informed participants that they would read syllogisms about different gene editing techniques and listed all of the techniques, so that participants knew the target words in the spatial task would refer to such techniques (e.g. Talen, CRISPR/Cas9). Participants were then in each trial asked to read a syllogism and think about whether it made sense or not without immediately giving a response. They were instructed to give their response only at the end of the trial, that is after completing the spatial task. After reading the instructions, they completed two practice trials where they received feedback on whether they had correctly judged the presented syllogisms. Then, they completed experimental trial blocks with no feedback provided. Four blocks of trials were administered, whereby the identical set of 10 syllogisms was presented in a new random order, in each block. Thus, with four replications, participants were exposed to 40 trials in total. Participants could take a break in between the blocks. At the end, participants reported their age, gender and how fluent they were in English. The experiment took approximately 30 min.

### 2.2. Results and discussion

We analysed data across all experiments using R Statistical Software [57]. We estimated mixed-effects model with lm4 package (glmer function [58]); for binary dependent variables (logistic regression models) and afex package (mixed function [59]); for factorial design and continuous dependent variables. Under each analysis, we present each model structure. For logistic regression models, we report estimated likelihood as descriptive statistics referred to as predicted probabilities.

#### 2.2.1. Accuracy of syllogism coherence ratings

First, we found that on 14% of trials participants judged coherence of the syllogisms incorrectly. Second, we estimated a mixed-model logistic regression (accuracy ~ 1 + coherence × psychological distance + [1 | participant], family = binomial[link = ‘logit’])^3^ with a significantly higher likelihood of responding accurately to coherent (predicted probability = 0.94, s.e. = 0.01) versus incoherent syllogisms (predicted probability = 0.83, s.e. = 0.02), OR [odds ratio] = 3.20, 95% CI [2.21, 4.62], *z* = 6.19, *p* < 0.001. The effect of psychological distance and psychological distance by coherence interaction were not significant, *p*s > 0.292. We removed the incorrectly judged trials from the analysis.

#### 2.2.2. Signal detection parameters

We present coding of Signal Detection parameters in table 4. The choice of coding is arbitrary and would lead to the same results regardless of the specific coding. For each participant, we computed the proportion of all the above types of responses. Based on these scores, we computed *c* and *d*′ parameters [55]. In our design (and across all experiments with the verticality design reported in this manuscript), higher *c* scores indicated a general bias towards selecting ‘below’ responses, while *d*′ indicated the extent to which participants were able to discriminate the correct spatial location of target words (table 5).

**Table 4.** Signal detection parameters across all experiments.

		participants’ response (of objective spatial position)	
		above/far	below/close
target’s presented spatial position	above/far	HIT	OMISSION
	below/close	FALSE ALARM	CORRECT REJECTION

**Figure 2.**
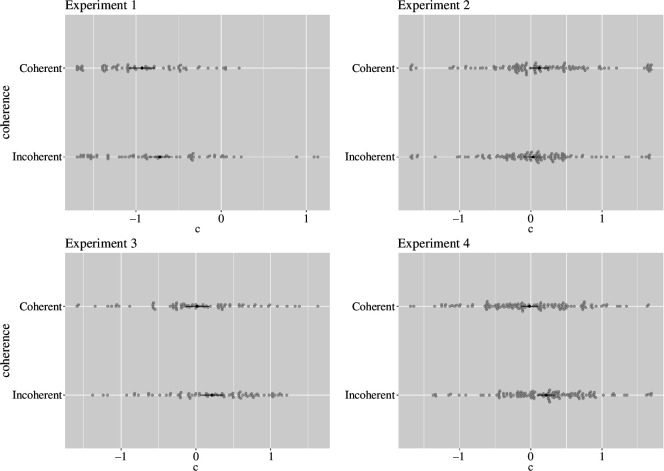
Response bias *c* as a function of coherence (Experiments 1, 3 and 4: higher *c* = stronger bias to respond below; Experiment 2: higher *c* = stronger bias to respond close).

We then subjected *c* parameters to a mixed model, with intercepts varying across participants (*c* ~ 1 + coherence × psychological distance + [1 | participant]). As hypothesized, we found that participants had a significantly higher tendency to select ‘below’ responses to incoherent (*M* = −0.72, s.e. = 0.08) versus coherent target words (*M* = −0.93, s.e. = 0.08), *t*(59) = 2.71, *p* = 0.009, Cohen’s *dz* = 0.35,^4^ 95% CI [0.09, 0.61] (see figure 2), suggesting that they associated coherent targets with the upper vertical position. The effect of psychological distance was not significant, *t*(59) = 0.13, *p* = 0.901, Cohen’s *dz* = 0.02, 95% CI [−0.23, 0.27], so was the interaction between coherence and psychological distance, *F*_1,59_ = 0.61, *p* = 0.440.

Further, we tested whether coherence affected participants’ ability to discriminate between the objective position of target words using *d*′ scores (*d*′ *~* 1 + coherence × psychological distance + [1 | participant]). We found that the effect was not significant, *t*(59) = 0.74, *p =* 0.463, Cohen’s *dz =* 0.09, 95% CI [−0.16, 0.35]. We also subjected *d*′ scores to a one-sample *t*‐test and found that there were no significant differences between the scores and 0, *t*(60) = 1.69, *p =* 0.096, Cohen’s *d =* 0.22, 95% CI [−0.03, 0.47] (*M =* 0.10, s.e. *=* 0.06).

#### 2.2.3. Exploratory analysis of incorrect responses

As on 14% of trials participants judged coherent syllogisms as incoherent, and incoherent syllogisms as coherent, we were interested to test whether the subjective perception of coherence, as determined by participants themselves and not by our materials, would also represented spatially. We subjected *c* parameters to a mixed model using the trials where participants made incorrect judgements. We found that the effect of coherence was significant, such that when participants judged coherent syllogisms as incoherent, they had a stronger bias to respond ‘below’ (*M =* −0.17, s.e. *=* 0.08) than when they judged incoherent syllogisms as coherent (*M =* −0.50, s.e. *=* 0.06), *t*(51) = 3.37, *p <* 0*.*001, Cohen’s *dz =* 0.44, 95% CI [0.17, 0.71].

#### 2.2.4. Objective spatial position

**Figure 3.**
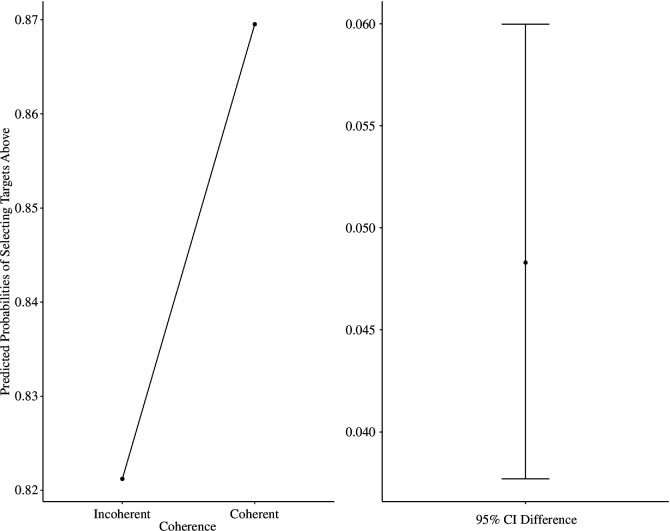
Predicted probabilities (estimated likelihood) of selecting targets ‘above’ as a function of coherence (Experiment 1).

To account for the potential effect of objective spatial position of the target words and to support the SDT (Signal Detection Theory) findings further, we estimated a mixed model logistic regression (with intercepts varying across participants), whereby we entered objective spatial position and coherence as fixed effects, with ‘above’ and ‘below’ responses as a binary outcome (above = 1 versus below = 0). We controlled for the psychological distance factor only, as it did not have an effect on biases to respond below in the signal detection analysis (location choice ~ 1 + coherence × objective spatial position + psychological distance + [1 | participant], family = binomial(link = ‘logit’). We found that the effect of coherence was significant, such that participants had a higher tendency to select ‘above’ responses for coherent (predicted probability = 0.87; s.e. = 0.02) versus incoherent targets (predicted probability = 0.82, s.e. = 0.03), OR [odds ratio] = 1.45, 95% CI [1.07, 1.95], *z* = 2.42, *p* < 0.001. This suggested that participants were 1.45 more likely to select ‘above’ responses to coherent versus incoherent targets (see figure 3). The main effect of psychological distance, position and coherence by position interaction were not significant, *p*s > 0.686.^5^

Our primary aim in Experiment 1 was to determine whether coherence was represented on the vertical dimension. We also aimed to assess whether coherence interacted with psychological distance. We found support for the hypothesis concerning verticality. Participants had a higher response bias to select upper vertical positions for targets that came from coherent syllogisms than incoherent syllogisms. In addition, we found the same effect for responses where participants incorrectly judged whether the syllogisms made sense or not. When they incorrectly thought that incoherent syllogisms were coherent, they represented them in the upper location. These results hint at the possibility that subjective representation of making sense has a spatial correlate on the vertical dimension, regardless of objective validity. Further, we found that participants did not perform above chance level at discriminating the objective position of words, suggesting that discrimination of the objective position of the target words was difficult.

Overall, in Experiment 1, we found initial evidence suggesting that coherence is represented on the vertical dimension. We did not find support for our predictions concerning psychological distance, as this manipulation did not influence participants’ response bias for ‘above’ versus ‘below’ judgements. In the next experiment, we tested whether coherence can be also mapped onto spatial proximity.

## 3. Experiment 2: coherence as spatial proximity

In the follow-up experiment, we tested whether coherence can also be mapped onto spatial proximity insofar as the task instruction makes spatial proximity salient. We used a similar design as in Experiment 1, again including the three-dimensional picture [54] simulating spatial proximity in the spatial task. We presented the same syllogisms; however, we removed the references to psychological distance as to only manipulate coherence. We instructed participants to focus on spatial proximity instead of verticality, and to indicate whether target words taken from the presented syllogisms appeared far or close to the participant. If coherence is represented as proximity between two points on the screen [11], then we would expect a general tendency to respond ‘close’ rather than ‘far’ to coherent versus incoherent words, regardless of the objective spatial position of the words.

### 3.1. Method

#### 3.1.1. Participants

We recruited 117 students at a Dutch university; as most students were fluent in English (see table 2 for details), the experimental materials were presented in English. Four participants were excluded because they did not complete the experimental trials, leaving the total sample size at 113 participants. We conducted a power analysis in G*Power for a two-tailed paired *t*‐test, and effect sizes ranging from Cohen’s *dz* = 0.25–0.35, 80% power, α = 0.05, yielding an *N* of 67–128. We included a lower range of effect sizes compared to Experiment 1 because the current experiment involved participants completing the study online, and this might lead to a higher trial loss. Participants received course credits for taking part in the study.

#### 3.1.2. Design and materials

#### Coherence syllogisms and spatial task

We adapted the design from Experiment 1 with four modifications. First, we removed the references to psychological distance from the syllogisms, such that the syllogisms only consisted of three sentences. Hence, the only manipulated factor was coherence (within-participants). Second, we changed the instructions by asking participants to indicate spatial proximity of the target words rather than the vertical location. Third, participants used horizontally arranged keys on the computer keyboard (*d* and *j*) to indicate their responses in the spatial task (i.e. to avoid confusion with vertically positioned keys—arrows up and down). We counterbalanced the response options between-participants, such that in one condition, *d* corresponded to far responses and *j* to close responses, while in the other condition, *d* corresponded to close and *j* to far responses. Fourth, we conducted the study online.

#### Procedure

Participants completed the experiment on Pavlovia platform (pavlovia.org) that can host experiments online programmed in PsychoPy. First, participants completed two practice trials followed by four blocks of experimental trials (there were 10 trials in each block) as in Experiment 1. At the end, participants reported their age, gender and English fluency.

### 3.2. Results and discussion

#### 3.2.1. Accuracy of syllogism coherence ratings

First, across the whole sample of participants, we excluded 35 trials (0.01%) on which participants’ responses to the spatial task were missing. Second, 11% of the remaining trials were judged incorrectly. Participants judged coherent (predicted probability = 0.95, s.e. = 0.01) versus incoherent syllogisms more accurately (predicted probability = 0.91, s.e. = 0.02), OR = 2.09, 95% CI [1.70, 2.58], *z* = 6.91, *p* < 0.001 (accuracy~1 + coherence + [1 | participant], family = binomial[link = ‘logit’]). The incorrectly judged trials were removed from the analysis.

#### 3.2.2. Signal detection parameters

For consistency, we classified participants’ responses in a similar way to the coding from Experiment 1, such that we replaced the ‘above’ responses with ‘far’, and ‘below’ with ‘close’ (see table 4). Based on these parameters, higher *c* scores corresponded to a general bias towards selecting close responses. We conducted a mixed model with participants as random intercepts (*c* ~ 1 + coherence + [1 | participant]). We found that participants had a significantly higher tendency to choose close responses to coherent (*M* = 0.12, s.e. = 0.07) versus incoherent target words (*M* = 0.03, s.e. = 0.07), *t*(111) = 2.18, *p* = 0.031, Cohen’s *dz* = 0.21,^6^ 95% CI [0.02, 0.39], indicating that participants associated coherence with close spatial proximity (see figure 2, Experiment 2).

Subsequently, we found that the effect of coherence was not significant when it came to participants’ ability to discriminate the objective spatial position of target, as represented by *d*′ scores, *t*(112) = 1.44, *p =* 0.153, Cohen’s *dz =* 0.14, 95% CI [−0.05, 0.32] (*d*′ *~* 1 + coherence + [1 | participant]). Unlike in Experiment 1, in the current study, using a one-sample *t*‐test, we found that *d*′ scores were significantly different from 0, *t*(113) = 2.91, *p =* 0.004, Cohen’s *d =* 0.27, 95% CI [0.08, 0.46] (*M =* 0.12, s.e. *=* 0.04), indicating that overall participants performed above chance level at discriminating objective spatial positions of the target words.

#### 3.2.3. Exploratory analysis of incorrect responses

As in Experiment 1, we analysed participants’ inaccurate responses in judging the syllogisms whereby participants classified coherent syllogisms as incoherent, and incoherent syllogisms as coherent. We found that participants did not represent subjective perception of coherence (incorrectly judging incoherent syllogisms as coherent) closer to themselves (*M =* 0.05, s.e. *=* 0.05) than incoherent ones (*M =* −0.07, s.e. *=* 0.06), *t*(79) = 1.73, *p =* 0*.*088, Cohen’s *dz =* 0.16, 95% CI [−0.02, 0.35].

#### 3.2.4. Objective spatial position

**Figure 4.**
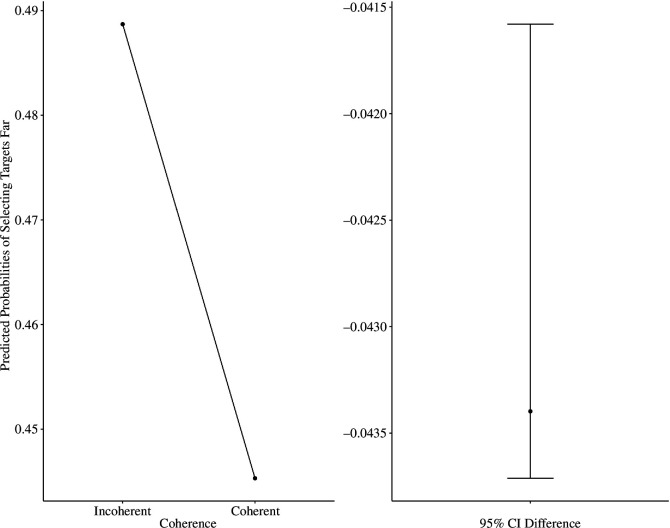
Predicted probabilities (estimated likelihood) of selecting targets ‘far’ as a function of coherence (Experiment 2).

To support the previous analyses, we also conducted mixed-model logistic regression like in Experiment 1 (location choice ~ 1 + coherence × objective spatial position + response mode [1 | participant], family = binomial(link = ‘logit’). First, we found that coherence significantly predicted the likelihood of selecting ‘far’ versus ‘close’ responses (coded as: 1—far, 0—close), with participants having a significantly higher tendency to select close spatial locations to coherent target words (predicted probability = 0.45; s.e. = 0.03) versus incoherent ones (predicted probability = 0.49, s.e. = 0.03), OR = 1.2, 95% CI [1.04, 1.37], *z* = 2.51, *p* = 0.011. This was true when controlling for response mode keys, which also did not have an effect on choices of spatial positions, *p* > 0.461. The main effect of position and the position by coherence interaction was not significant, *p*s > 0.188 (see figure 4).

To summarize, we found evidence for coherence being represented in close subjective spatial proximity, complementing the findings from earlier research on coherence being projected onto horizontal space [11]. Altogether, this evidence suggests that mental representation of coherence is malleable with regard to metaphorical mappings. In the next experiments, we aimed at replicating the findings from Experiment 1 using different designs and materials.

## 4. Experiment 3: coherence as verticality in abstract syllogisms

So far, we demonstrated a mapping of coherence representations onto the vertical dimension and subjective spatial proximity. However, the interpretation concerning verticality in Experiment 1 is still somewhat ambiguous inasmuch the experimental tool we used, that is the background drawing adapted from Bar-Anan *et al*. [54] was, in their research, not aimed at finding evidence for a vertical mapping at all. Rather, the drawing was aimed at an operationalization of spatial proximity, not verticality *per se*. By virtue of the graphic design, however, verticality was implied as being confounded with proximity: the three-dimensional perspective implied by the two-dimensional drawing meant that things in the upper half were to be seen as further away from the viewer than things in the lower half. This way, since a two-dimensional interpretation (verticality) and a three-dimensionalinterpretation (proximity) of the drawing are perceptually confounded in the stimulus, we needed a disambiguation in terms of a clearer demonstration that verticality itself was the crucial factor in the experiment.

Hence, in Experiment 3, we aimed at replicating these findings by designing a similar experiment to Experiment 1 with a few modifications. Specifically, we removed the template drawing of the pathway in the spatial task, and we did not refer to psychological distance in the materials. To further avoid potential confounds associated with the content of the syllogisms about gene editing, we used abstract syllogisms instead, with unrealistic content that, as per instruction, participants were supposed to take as describing phenomena in a fantasy world. We pre-registered the hypotheses, method, materials and analyses on OSF: https://doi.org/10.17605/OSF.IO/9ASQN.

### 4.1. Method

#### 4.1.1. Participants

Seventy participants from a British university took part in the study (see table 2 for details). We estimated the sample size based on a power analysis in G*Power for a paired and one-tailed *t*‐test, and the effect size that was slightly smaller than the one obtained in Experiment 1 (due to detecting a small effect size in Experiment 2): Cohen’s *dz* = 0.30, alpha = 0.05 and 80% of power (the power analysis was pre-registered). The analysis estimated 66 participants. Participants received course credits for their participation.

#### 4.1.2. Design and materials

#### Syllogisms

We manipulated coherence using a similar design to Experiment 1. To avoid confounds associated with content, such as believability, each syllogism described a fantasy syllogism about animals and plants. We used the following instructions for participants: ‘for all syllogisms, please imagine you are in a garden where genetic manipulation took place, such that normal biological rules do not apply. There might be unusual combinations of things and features, but just take it as science fiction. Your task will be to focus entirely on the logical structure of the syllogisms when making your decision whether they make sense or not’. There were two types of coherent and two types of incoherent syllogisms. Coherent syllogisms were either structured as ‘All A are B. All B are C. All A are C’ or as ‘Some A are B. All B are C. Some A are C’. Incoherent syllogisms were either structured as ‘All A are B. All B are C. All C are A’ or as ‘All A are B. Some B are C. Some A are C’. As in Experiment 1, the first two sentences in each syllogism were the premises (e.g. ‘All pears are sappy. All sappy fruits are aggressive’), while the last sentence was the conclusion (e.g. ‘All pears are aggressive’.). Each participant saw the same set of five coherent and five incoherent syllogisms in a new random order, in each block of trials (see table 5 for all syllogisms).

**Table 5.** Materials based on form in Experiment 3.

coherent	target word
All pears are sappy.	aggressive pears
All sappy fruits are aggressive.	
All pears are aggressive.	
Some strawberries are blue.	happy strawberries
All blue fruits are happy.	
Some strawberries are happy.	
Some snails live in the water.	winged snails
All animals living in the water have wings.	
Some snails have wings.	
All butterflies like sunny weather.	lazy butterflies
All animals liking sunny weather are lazy.	
All butterflies are lazy.	
Some plums are sweet fruits.	sticky-legged plums
All sweet fruits have sticky legs.	
Some plums have sticky legs.	

Incoherent	target word
All fruits are ripe	cubic fruits
All ripe fruits are cubic eggs.	
All cubic eggs are fruits.	
All healthy fruits are tiny fruits.	healthy mammal fruits
All tiny fruits are mammals.	
All mammals are healthy fruits.	
All raspberries grow on trees.	unhealthy raspberries
Some fruits growing on trees are unhealthy.	
Some raspberries are unhealthy.	
All birds with four wings are at the lake.	birds geese
Some birds at the lake are geese.	
Some birds with four wings are geese.	
All peaches are soft.	warm-blooded peaches
Some soft fruits are warm-blooded.	
Some peaches are warm-blooded.	

#### Spatial task

After reading a syllogism in each trial, participants completed a similar spatial task as in Experiment 1. Because in this task, we removed the picture of the pathway, we believed the task was slightly more difficult than in the previous experiment. To deal with this, we increased the presentation duration of target words from 200 to 500 min. We first presented participants with a grey circle followed by a target expression taken from the syllogism presented at the beginning of the trial (e.g. aggressive pears). Participants’ task was to quickly and accurately judge whether the target expression was presented ‘above’ or ‘below’ the midpoint of the circle presented earlier (verticality instruction). At the end of each trial, participants indicated whether the syllogism made sense or not (see figure 5 for details). The size and vertical location of stimuli were the same as in Experiment 1.

**Figure 5.**
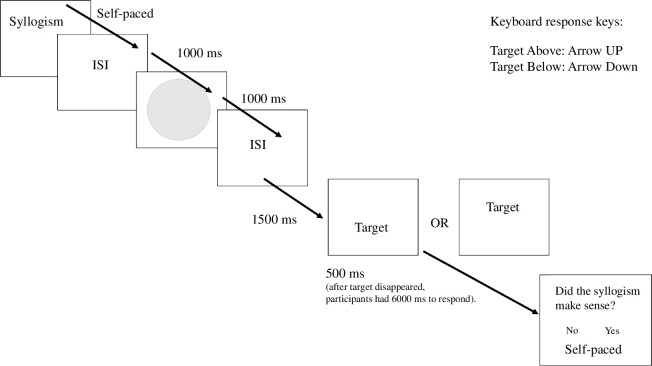
Trial events and timing (min) in Experiment 3.

#### Procedure

The procedure was the same as in Experiment 1, except the experimenter provided participants with training (four additional practice syllogisms) on how to judge the validity of a syllogism. We did this because of the increased task difficulty in terms of using formal syllogisms with unrealistic content. Participants completed two practice trials before starting the main task.

### 4.2. Results and discussion

#### 4.2.1. Accuracy of syllogism coherence ratings

Participants judged syllogisms incorrectly on 24% of trials, with a significantly higher likelihood of responding accurately to coherent (predicted probability = 0.95, s.e. = 0.01) than incoherent syllogisms (predicted probability = 0.63, s.e. = 0.03), OR = 10.72, 95% CI [8.42, 13.65], *z* = 19.24, *p* < 0.001 (accuracy ~ 1 + coherence + [1 | participant], family = binomial[link = ‘logit’]). As in the previous study, we deleted incorrectly answered trials from the analysis.^7^ We note that it is possible that some incoherent syllogisms might have appeared coherent to participants. Even though such syllogisms (e.g. All peaches are soft. Some soft fruits are warm-blooded. Some peaches are warm-blooded) were not deductively coherent, they might have appeared coherent when using inductive reasoning. This potentially explains lower accuracy scores for incoherent syllogisms.

#### 4.2.2. Signal detection parameters

We computed signal detection parameters and conducted the same type of analyses as in Experiment 1. Using *c* parameters (*c ~* 1 + coherence + [1 | participant]), we again found that participants showed a significantly stronger bias to select ‘below’ responses for incoherent (*M =* 0.21, s.e. *=* 0.08) than coherent target words (*M =* 0.02, s.e. *=* 0.08), *t*(69) = 2.40, *p =* 0*.*019, Cohen’s *dz =* 0.29,^8^ 95% CI [0.05, 0.53] (see figure 2, Experiment 3), suggesting that with the graphic elements (narrowing pathway background) confounding verticality with three-dimensional proximity removed, participants still showed a bias in associating coherence with upper vertical positions, replicating and extending the results from Experiment 1. Now, we obtained more unambiguous support for the hypothesis of verticality being a physical target dimension of coherence, via metaphorical mapping.

Further, we examined participants’ ability to discriminate the objective position of target words. We found no significant differences in their discrimination ability between coherent and incoherent targets using *d*′ scores (*d*′~1 + coherence + [1 | participant]), *t*(69) = 1.12, *p* = 0.267, Cohen’s *dz* = 0.13, 95% CI [−0.10, 0.37]. However, across all trials, participants were above chance level (i.e. greater than 0) at discriminating the objective position of the target words, *t*(69) = 3.28, *p* = 0.002, Cohen’s *d* = 0.39, 95% CI [0.15, 0.63] (*M* = 0.26, s.e. = 0.08). These findings are also in line with the results from the previous experiment.

#### 4.2.3. Exploratory analysis of incorrect responses

As in Experiment 1, we tested whether participants had a stronger bias to select ‘below’ responses for coherent targets judged as incoherent than incoherent targets judged as coherent. However, the difference was not significant, *t*(59) = 1.37, *p =* 0.177, Cohen’s *d =* 0.17, 95% CI [−0.07, 0.41], (incoherent targets judged as coherent: *M =* −0.05, s.e. *=* 0.06; coherent targets judged as incoherent: *M =* 0.08, s.e. *=* 0.08). These findings suggest that in case of incorrect responses, participants’ subjective judgements of coherence in the case of abstract syllogisms did not translate into spatial mental representation. As the validity of the syllogisms was probably more difficult to judge (as seen in the high number of inaccurate trials) than the materials in Experiment 1, it is possible that participants’ mistakes reflected confusion or misunderstanding of the syllogisms, rather than confident, subjective judgements about coherence in the syllogisms. Hence, overall, such misunderstanding in contrast to meaningful processing was not significantly supported by spatial processing.

#### 4.2.4. Objective spatial position

As in Experiment 1, we examined whether participants were more likely to respond ‘above’ to coherent than to incoherent targets using a mixed-model logistic regression analysis 001 (location choice ~ 1 + coherence × objective spatial position + [1 | participant], family = binomial(link = ‘logit’). As hypothesized, participants were significantly more likely to select ‘above’ responses for coherent (predicted probability = 0.49; s.e. = 0.03) versus incoherent targets (predicted probability = 0.41, s.e. = 0.03), OR = 1.32, 95% CI [1.001, 1.74], *z* = 1.97, *p* = 0.049 (see figure 6). We then investigated whether coherence interacted with objective spatial positions of target words. The interaction was not significant, OR = 1.14, 95% CI [0.77, 1.68], *z* = 0.66, *p* = 0.508. In addition, we found that the effect of objective spatial position was significant, OR = 1.59, 95% CI [1.15, 2.11], *z* = 2.88, *p* = 0.004, whereby participants had a higher tendency to respond ‘above’ to target words presented above the midpoint (predicted probability: 0.51, s.e. = 0.03) than to targets presented below the midpoint (predicted probability: 0.39, s.e. = 0.03).

**Figure 6.**
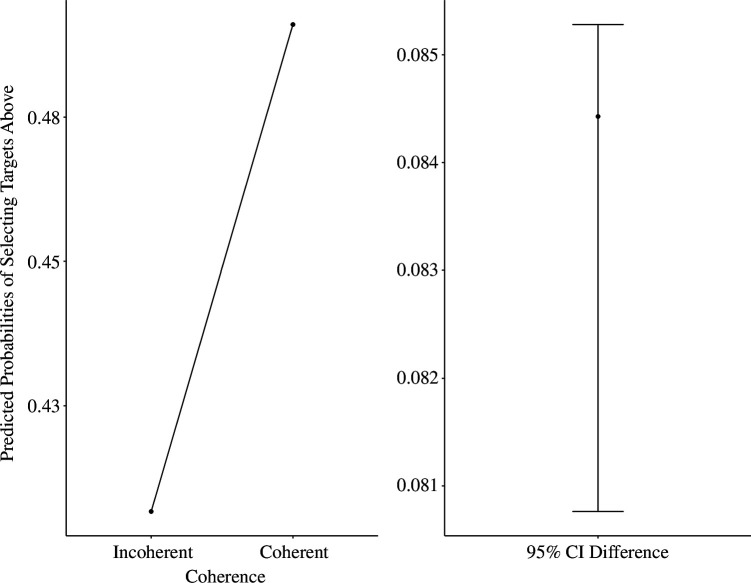
Predicted probabilities of selecting targets ‘above’ as a function of coherence (Experiment 3).

To conclude, we fully replicated the findings from Experiment 1 using different materials (abstract syllogisms), suggesting that coherence is represented on the vertical spatial dimension. We designed Experiment 4 to replicate these findings in a different population of participants, and as in Experiment 1 using gene editing syllogisms to manipulate coherence.

## 5. Experiment 4: coherence as verticality in realistic syllogisms

In Experiments 1 and 3, we found evidence for spatial correlates of coherence on the vertical dimension. To further replicate these findings, we aimed at recruiting a population of participants in The Netherlands. We used the materials from Experiment 1 and the design (presentation modalities) from Experiment 3. We conducted the experiment online using Pavlovia. We hypothesized that participants would represent coherence in the upper vertical location. We pre-registered the hypothesis, method, materials and analyses on OSF: https://doi.org/10.17605/OSF.IO/XWCES.

### 5.1. Method

#### 5.1.1. Participants

We recruited 118 participants from a population of university students in The Netherlands using materials in English (see table 2 for details). We conducted the same power analysis as in Experiment 2. The analysis was consistent with our pre-registration and the analysis parameters were set to a two-tailed paired *t*‐test, effect size of Cohen’s *dz* = 0.28,^9^ 80% power and α = 0.05. The effect size was based on the mean effect size obtained in the previous studies. The estimated sample size was 115. Participants received course credits for taking part in the study.

#### 5.1.2. Design and materials

**Figure 7.**
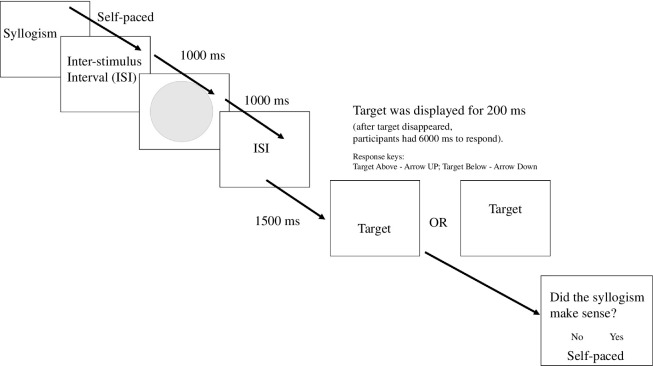
Trial events and timing (min) in Experiment 4.

We used the same syllogisms as in Experiment 1. We manipulated coherence within-participants as in the previous studies. We adapted the design from Experiment 3, i.e. the presentation mode without the picture of a pathway simulating spatial proximity see figure 7 for details).

#### 5.1.3. Procedure

We conducted the study online. As previously, there were two practice trials and 40 experimental trials. After the task, participants reported their age, gender and English fluency. The experiment took around 30 min.

### 5.2. Results and discussion

#### 5.2.1. Accuracy of syllogism coherence ratings

We found that participants were incorrect in judging validity of the syllogisms on 14% of trials. As in previous experiments, participants were significantly more accurate to judge coherent (predicted probability: 0.93, s.e. = 0.01), versus incoherent syllogisms (predicted probability: 0.88, s.e. = 0.01), OR = 1.92, 95% CI [1.61, 2.30], *z* = 7.13, *p* < 0.001 (accuracy ~ 1 + coherence + [1 | participant], family = binomial[link = ‘logit’]). As in previous studies, we removed the inaccurate trials from the analysis.

#### 5.2.2. Signal detection parameters

As hypothesized, using *c* parameters, we found a significantly stronger bias towards selecting ‘below’ responses for incoherent (*M =* 0.18, s.e. *=* 0.06) than coherent target words (*M =* 0.03, s.e. *=* 0.06), *t*(117) = 2.84, *p <* 0*.*001 (*c ~* 1 + coherence + [1 | participant]), Cohen’s *dz =* 0.26, 95% CI [0.08, 0.45] (see figure 2, Experiment 4), replicating the results from Experiments 1 and 3.

Subsequently, we found that participants’ discrimination ability (*d*′) did not vary as a function of coherence, *t*(117) = 1.45, *p* = 0.149, Cohen’s *dz* = 0.13, 95% CI [−0.05, 0.31] (*d*′ ~ 1 + coherence + [1 | participant]). Finally, across both types of trials, participants were above chance level (i.e. greater than 0) when discriminating the spatial position of target words, *t*(117) = 5.35, *p* < 0.001, Cohen’s *d* = 0.49, 95% CI [0.30, 0.68] (*M* = 0.25, s.e. = 0.05).

#### 5.2.3. Exploratory analysis of incorrect responses

As in previous studies, we were interested to see whether participants had a tendency to represent incoherent targets in the upper visual field (above the imagined midpoint) on trials where they made a mistake and considered them as coherent. Indeed, this was the case: in terms of *c* parameters, we found that participants had a significantly stronger bias to select objectively incoherent targets ‘above’ when they judged them as coherent (*M =* −0.05, s.e. *=* 0.05) than objectively coherent targets when they thought they were incoherent (*M =* 0.17, s.e. *=* 0.05), *t*(88) = 3.54, *p <* 0.001, Cohen’s *d =* 0.34, 95% CI [0.15, 0.54], replicating the results from Experiment 1, but not Experiment 3, where we had used abstract syllogisms.

#### 5.2.4. Objective spatial position

**Figure 8.**
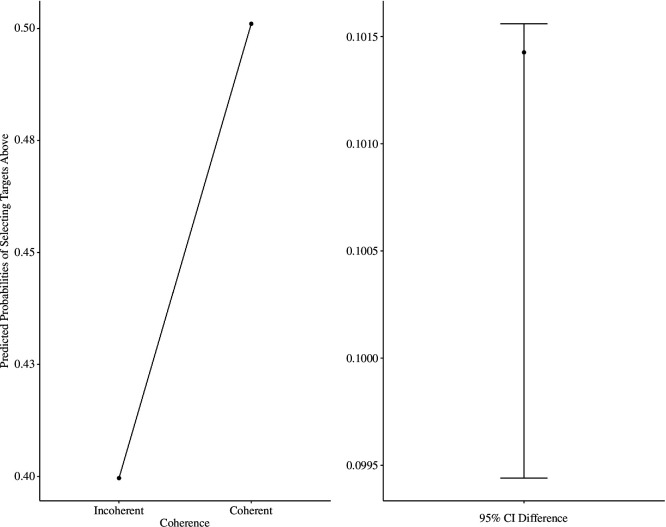
Predicted probabilities of selecting targets ‘above’ as a function of coherence (Experiment 4).

We conducted the same analyses as in Experiments 1 and 3 (location choice ~ 1 + coherence × objective spatial position + [1 | participant], family = binomial(link = ‘logit’). As predicted, participants had a higher tendency to select ‘above’ responses for coherent (predicted probability = 0.48; s.e. = 0.02) versus incoherent targets (predicted probability = 0.42, s.e. = 0.02), OR = 1.46, 95% CI [1.22, 1.74, *z* = 4.08, *p* < 0.001 (see figure 8). We also found that the effect of objective spatial position was significant, OR = 1.76, 95% CI [1.47, 2.11], *z* = 6.97, *p* < 0.001, whereby participants had a higher tendency to respond ‘above’ to target words presented above the midpoint (predicted probability: 0.51, s.e. = 0.02) than to targets presented below the midpoint (predicted probability: 0.40, s.e. = 0.02). The objective position by coherence interaction was not significant, *p* = 0.075.

Again, these findings are consistent with the ones obtained in Experiments 1 and 3 and provide further support for the previously found pattern in *c* parameters, demonstrating that reasoning about coherence involves spatial processing on the vertical dimension.

## 6. Experiments 1–4: exploratory analysis of reasoning accuracy and spatial mapping

In light of previous theorizing that spatial mechanisms support reasoning in general [11, 38,60], we aimed at examining whether reasoning accuracy was associated with spatial representation. Specifically, we conducted an exploratory analysis to test whether selecting spatial cues that are semantically linked to coherence (above/close) while reasoning was correlated with a higher probability of judging the validity of a syllogism correctly. We predicted that participants would be more accurate in judging coherent syllogisms if the selected response in the spatial task was the upper/close versus lower/far location, while the opposite would be true for incoherent syllogisms: participants’ performance would be more accurate when selecting the lower/far versus upper/close location.

### 6.1. Results and discussion

To test this prediction, we combined the data across all experiments (*n* = 362). In Experiment 2, we previously examined the mapping of coherence onto spatial proximity rather than verticality like in the other experiments. Hence, we recoded the spatial task responses in Experiment 2. As we hypothesized that there would be an accuracy advantage when selecting upper and close spatial locations for coherent syllogisms, and lower and far spatial locations for incoherent syllogisms, we recoded the close and far responses into ‘above’ and ‘below’ locations, respectively, in the analysis.^10^

**Figure 9.**
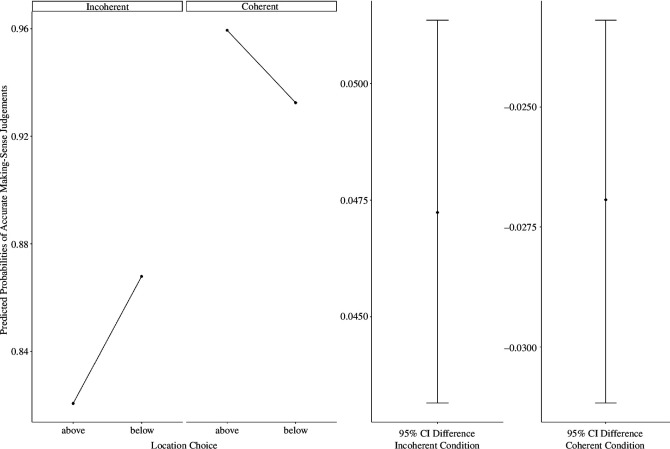
Predicted probabilities of response accuracy in solving syllogisms (all experiments).

To account for having multiple experiments in a single analysis, we conducted a mixed-model logistic regression with participants nested within experiments. We specified syllogistic judgement accuracy scores as the outcome (0— inaccurate, 1—accurate), and syllogism coherence (coherent versus incoherent) and location choice (above-close/below-far) as predictors: accuracy ~ 1 + coherence × location choice + [1 | participant: experiment], family = binomial(link = ‘logit’). As hypothesized, we found that the interaction between syllogism coherence and location choice was significant, OR = 2.46, 95% CI [1.98, 3.05], *z* = 8.12, *p* < 0.001, such that accuracy in judging coherent syllogisms was significantly higher when participants selected the above/close position (predicted probability: 0.96, s.e. = 0.01) than below/far position (predicted probability: 0.93, s.e. = 0.01), OR = 1.71, 95% CI [1.43, 2.05], *z* = 5.79, *p* < 0.001. For incoherent syllogisms, we found the opposite pattern: accuracy was higher when participants selected the below/far (predicted probability: 0.87, s.e. = 0.01) than above/close position (predicted probability: 0.82, s.e. = 0.01), OR = 1.44, 95% CI [1.26, 1.64], *z* = 5.79, *p* < 0.001 (see figure 9). These results suggest that spatial representation of coherence is linked with accuracy in reasoning performance. Future research should explore whether spatial representation and accuracy in reasoning are causally related.

## 7. General discussion

Making sense is one of the key human needs [25]. People aim to perceive elements in the world to have expected and consistent relations, and such coherence represents comprehension of the world and a key facet of meaning in life [26]. In the present research, we examined the idea that reasoning about coherence—a highly abstract experience—is represented using concrete spatial dimensions insofar as such dimensions can be meaningfully used in processing within a given context.

Across three experiments, participants showed a response tendency to see a target expression taken from a coherent syllogism as located at a high spatial position, and an expression from an incoherent syllogism at a low spatial position. This tendency was independent of the true position of the target and consistent across different types of materials: syllogisms based on content or strict logical form of the premises. Coherence simulations at a high versus a low spatial level were even demonstrated for incorrectly answered trials (Experiments 1, 3 and 4), supporting the idea that response generation during the spatial task did indeed follow a self-generated or subjective spatial representation of coherence, thereby replicating an analogous finding in previous research [11].

Importantly, using the same presentation modality with a three-dimensional pathway simulating spatial proximity as in Experiment 1 but with modified instructions focused on spatial proximity rather than verticality, we observed a tendency to represent coherence in spatial closeness to oneself (Experiment 2). Participants showed a stronger bias to select coherent versus incoherent targets in spatial closeness to themselves, suggesting that depending on the task instruction, coherent elements are not only represented in spatial proximity of two objects in horizontal space, but they can also be mapped onto spatial proximity in relation to one’s own physical position. Overall, in all experiments, except Experiment 1, participants’ accuracy vis-à-vis the objective (i.e. the actual) spatial location of the targets was above chance, irrespective of whether the target came from a coherent or from an incoherent syllogism. In addition, we also provide correlational evidence for the idea that spatial representations in abstract thinking are related to being accurate in logical reasoning.

### 7.1. Malleability in the representation of abstract concepts

Our evidence demonstrates malleability in the spatial representation of coherence on the vertical dimension and subjective proximity to oneself, posing a challenge to embodied perspectives of cognition. Previous literature postulated that there are likely *a priori* acquired metaphoric links (derived from sensorimotor experiences) that underlie associations between a particular abstract concept and a specific spatial dimension (e.g. power is tied to verticality [10], coherence is tied to spatial proximity [11]). Our findings demonstrate that the cognitive system adapts to the available format of projection: when the setting affords a vertical representation, then an abstract concept would be mapped onto that dimension, but when the setting affords a horizontal representation, that dimension would be used. This is true even though some spatial mappings might be less intuitive than others: it is more intuitive to assume that concepts that make sense together, also fit together and hence are represented close to each other [11], rather than at an elevated height.

In the present studies, we show that even a less intuitive mapping, i.e. verticality, can be made efficient in processing, as soon as the context (i.e. the task instruction) makes that mapping available. We therefore suggest that space is a malleable toolbox where different spatial dimensions can be utilized online for reasoning in adaptive ways depending on the task/environmental demands. As such, spatial dimensions used in mental representations are unlikely to be metaphors, but metaphoric meaning is acquired after a flexible allocation of a particular task-related concept to space is completed (e.g. coherence to proximity or coherence to vertical height; see §7.2 for how particular cues are likely linked to a particular concept). Possibly, such acquisition is based on learning history.

More specifically to coherence, we believe that our findings also take a step forward from other recent findings on metaphorical mappings. An earlier paper [11] examining the nature of spatial metaphors underpinning the experience of coherence indicated a mapping of coherence onto proximity: whatever was experienced as ‘making sense’ (*balance* within Heiderian triads, *validity* in categorical syllogisms, *latent causality* in small scenarios) appeared to be represented in mental space such that the concepts involved were closer together in coherent cases, as compared with incoherent cases. As an immediate critique of the paper, however, the question comes to mind to what extent this mapping, despite its undeniable plausibility, may still be (partly) caused by experimental circumstances, that is the particularities of the design that had been used. In this respect, one had to acknowledge not only that there exist other even though less intuitive mappings, but more generally, that the cognitive system might be as flexible as *not* to create fixed links between a particular abstract concept on one side and a particular physical dimension on the other. From this, one had to be open to a more episodic analysis of the mapping process itself.

Indeed, attentional processes might guide the construction of episodic models in working memory, and the nature of these processes possibly determines the reliance on one particular spatial mapping that might result from model construction [50,52,61]. As such, the mapping process would appear rather malleable and flexible, depending on situational constraints. For example, the spatial modelling of experienced time (as self-moving versus time-moving) depended on the focus one had on one’s actual spatial situation [62]. During a train ride, they found more self-moving representations at the beginning and the end of the journey, whereas in the middle of it, the same tendency was much less prominent. In other words, an external cue contributed to the nature of the mapping of time onto space. Metaphoric mapping is also susceptible to experimental constraints and instructions. Metaphorically grounded spatial effects may be present or absent depending on whether participants’ attention is drawn to particular task dimensions [48]. Ultimately, specific mappings are likely being formed in working memory and are of episodic nature, rather than hard-wired and linked between abstract and physical domains in long-term memory [16,50,63,64] for a linguistic analysis, see [65,66].

In this light, we challenge previous results [11]. In Experiment 1, the stimulus material (i.e. the template drawing) gave opportunity for both types of mapping: proximity by means of a three-dimensional interpretation of the drawing, and verticality by means of a two-dimensional interpretation. As the instruction for the spatial task, however, clearly made verticality salient, we predicted and found a vertical mapping, this way ruling out a fixed, long-term memory-based link between coherence and proximity. Psychological distance, as additionally manipulated in this experiment, had no spatial effect, although a mapping onto proximity had been demonstrated by Bar-Anan *et al*. [54] from where the template drawing had originated. We submit that different from theirs, our task instruction (with respect to locating the target words) directed participants’ attention uniquely to the vertical dimension. This part of the instruction then dominated the process in terms of exclusively focusing attention on the words’ meaning (in terms of coherence versus incoherence) in relation to their position on the screen, with psychological distance information likely not being processed. In contrast, in Experiment 2, we showed that directing attention to spatial proximity cues resulted in coherence being mapped onto spatial closeness to oneself. This further strengthens the idea that attentional processes likely guide mental representation of abstract concepts and the links between space and concepts are not fixed in long-term memory.

### 7.2. Semantic spatial cues

It appears that in order to elicit mapping phenomena of the described sort, the spatial constraints set by the experimental task have to carry the potentiality to be linked to semantic elements within the materials that will allow a plausible spatial meaning or ‘blending’ [67] to be related to the relevant spatial dimension [48]. In our case, as explained in §1, there are several ways of establishing such links between coherence and verticality: (i) abstractness of the coherence experience (connected mainly with the conclusion) vis-à-vis the basic pieces of information (contained in the premises) within syllogisms [7]; (ii) greater ‘overview’ over a set of propositions from an assumed or simulated ‘high’ position [36]; or (iii) more positive connotations of ‘high’ (as opposed to ‘low’) in combination with more positive connotations of ‘valid’ (as opposed to ‘invalid’) [17,40]. Another possibility is that coherence acquires a verticality representation through embodied grounding in social interactions [6,68]. Indeed, the acquisition of meaning, especially in terms of higher-level logical reasoning, is developed usually through asymmetrical social interactions whereby more knowledgeable and powerful others (teachers) fill in learners’ knowledge gaps. For this reason, understanding complex abstract relations may in fact be socially embodied or grounded in social interactions.

Yet, in this article, we do not strive at establishing a causal link between any of these three possibilities and the observed vertical bias for coherence. Rather, our findings imply a link between task conditions, inclusive of type of instruction, and the nature of the mapping process (verticality and spatial proximity in relation to oneself as manipulated instruction, and the ensuing observed mapping), in comparison with the earlier results [11]. As a corollary, we submit that when the cognitive system finds a semantic link between the salient spatial dimension and the abstract concept (see above: (i), (ii) or (iii), or maybe still something else), it will use that salient dimension for a spatial mapping. Once such spatial mapping occurs, an abstract concept becomes metaphorically associated with a particular spatial position.

### 7.3. Implications for reasoning and future research

We additionally provide correlational evidence demonstrating that the probability of accurate judgements in the logical reasoning is linked to selecting a semantically spatial cue relevant to coherence (above/close) or low coherence (below/far). Participants were more likely to be accurate in judging the syllogism when they also represented coherence in the upper visual field of the vertical dimension or as close to themselves (i.e. using the semantically relevant cues), in contrast to the lower visual field and far away. The opposite was true when selecting cues associated with low coherence: participants were better at accurately judging invalid syllogisms when choosing below/far spatial cues. Future research should experimentally assess whether the relationship between spatial representation and accuracy in reasoning is also causal.

These results may also have implications for belief formation beyond logical reasoning. Theorists suggest that as abstract concepts are more difficult to understand than concrete concepts, people monitor abstract knowledge to verify its reliability in light of previous knowledge and new evidence [69]. Such knowledge is acquired primarily through interactions with others, and this enables the development of the social metacognition mechanism. That is, people are aware of their conceptual knowledge and the need to verify by consulting this knowledge with others. When examining scientific concepts associated with the COVID-19 pandemic (e.g. herd immunity), it has been found that people are less confident about their own beliefs about such concepts, and this is also accompanied by decreased confidence in scientists’ knowledge [70]. As such, it is likely that while evaluating highly abstract information, using spatial mental representations relevant to the reasoned abstract information might increase confidence in the information and its acceptance. For example, perceiving concepts in a close spatial location to oneself combined with previous positive attitudes towards the concept might be associated with increased acceptance of that concept as true. Indeed, indirect evidence for this is that subjective perceptions of psychological distance (i.e. imagining spatial, temporal, social and hypothetical closeness) to science has been found to predict more positive attitudes towards science [71].

Further, there is a possibility that semantic content associated with spatial distance, such as coming here/going away of an object discussed in a syllogism, would interact with formal logic in the representation of making sense. That is, the experience of formal logic should be more likely associated with spatial closeness when the conclusion of a syllogism refers to an object coming close versus further away. We believe that this is likely as long as participants’ attention in the task instructions is directed towards both formal logic and semantic content. Future research should examine this possibility directly.

## 8. Conclusion

We examined whether the mental representation of coherence is malleable and can employ different spatial mappings. We found that the experience of coherence is not grounded in a single, fixed in long-term memory link to horizontal spatial proximity. Instead, we show that directing people’s attention to a particular spatial cue is likely associated with the cognitive system employing that cue in representing coherence. As such, we show evidence for coherence being represented in upper location of the vertical dimension as well as in subjective spatial closeness to oneself. Such spatial representations were connected to being accurate when making validity judgements. To conclude, spatial representation of coherence is malleable, suggesting that space does not have *a priori* metaphoric links tied to abstract concepts. Rather, it represents a mental toolbox that can be flexibly utilized in cognition. Metaphoric links are only acquired *after* a particular (semantically relevant) spatial cue is allocated to an abstract concept.

## Data Availability

The data is accessible at [72].

## Footnotes

^1^
By spatial proximity, we refer to the spatial dimension, which can imply high spatial distance as well as high proximity.

^2^
For example, a balanced triad refers to a situation where two people like each other and dislike the third person (high coherence), while an unbalanced triad is where two people dislike each other, but they like the third person (low coherence).

^3^
Across all experiments, we also tested model fit when adding stimuli as random intercepts. The results remained largely the same.

^4^
The power analysis was based on a range of effect sizes: *dz =* 0.35–0.40.

^5^
The objective spatial position refers to the actual position of target words presented on the screen rather than participants’ judgements about the targets’ position.

^6^
The power analysis was based on a range of effect sizes: Cohen’s *dz* = 0.25–0.35.

^7^
We pre-registered different exclusion criteria than the ones used in the final analysis. Before data collection, we believed that it would be likely that a significant number of participants might not understand the structure of abstract syllogisms and hence judge them incorrectly. Based on this assumption, we aimed at excluding participants with accuracy scores at a chance level (50%). However, after obtaining data, we noticed that only two participants needed to be excluded due to this criterion without capturing a large number of incorrect responses. We found that participants found the abstract syllogisms difficult to judge (24% of all trials were judged incorrectly, that is 10% more than in Experiment 1). Therefore, to remove noise and to be consistent with the Experiment 1 exclusion criterion, we deleted all the incorrect trials.

^8^
The power analysis was based on Cohen’s *dz* = 0.30.

^9^
The power analysis was based on Cohen’s *dz* = 0.28.

^10^
We obtained the same results pattern when we excluded Experiment 2 from the analysis.
